# Characteristics of Two-Year College Students on the Autism Spectrum and Their Support Services Experiences

**DOI:** 10.1155/2015/391693

**Published:** 2015-11-15

**Authors:** Anne M. Roux, Paul T. Shattuck, Jessica E. Rast, Julianna A. Rava, Amy D. Edwards, Xin Wei, Mary McCracken, Jennifer W. Yu

**Affiliations:** ^1^A.J. Drexel Autism Institute, Drexel University, 3020 Market Street, Suite 560, Philadelphia, PA 19104-3734, USA; ^2^SRI International, 333 Ravenswood Avenue, BS 162, Menlo Park, CA 94025-3493, USA

## Abstract

Approximately 80% of college-going youth with autism in the US attend a 2-year college at some point. These community-based, universally accessible institutions offer both academic and vocational courses and have experience in teaching diverse learners. This study used nationally representative survey data from the National Longitudinal Transition Study-2 to describe the characteristics and services experiences of adults with autism who attended postsecondary education after high school, focusing on those who attended a 2-year college. Over 60% of those who attended 2-year colleges had little to no trouble conversing or performing functional skills like counting change during high school, and extracurricular participation was common (93.8%). Most 2-year college attenders (85.7%) were able to navigate to places outside the home versus 43.9% of those with no postsecondary education. Over half took vocational courses at 2-year colleges, while one-quarter pursued academic study. Less than half (48.6%) of those who disclosed their disability to the school reported receiving services, accommodations, or other help. Most (87.3%) felt they received enough help, but fewer (68.0%) felt the services they received were useful. Future research should delineate specific needs of students with autism in 2-year college settings and identify what supports are needed to improve persistence and completion rates.

## 1. Introduction

The reported prevalence of autism has been increasing for over a decade. Most of the increasing prevalence is among children with average to above-average intelligence [1]. Approximately one-third of youth on the autism spectrum in the United States go to college within eight years of exiting high school [2, 3], and college attendance is projected to increase in this population [4]. Yet, very few population-based studies are available that describe the experiences of young adults on the autism spectrum who attend college.

Two-year colleges are a primary gateway to postsecondary education for youth on the autism spectrum in the US. The term “2-year colleges” primarily refers to over 1,100 locally funded community colleges throughout the US [5], which are attended by about 40% of undergraduate students [6]. These universally accessible, open-admission educational institutions offer both academic and vocational courses and are experienced in teaching diverse learners who may require developmental academic support.

Of youth on the autism spectrum in the US who attend college, the vast majority (81%) attend a 2-year college either solely or as a stepping stone to a 4-year college [7]. Approximately 70% of 2-year public institutions in the US report enrolling students with autism spectrum disorders [8]. However, little research has specifically examined the characteristics of 2-year college students on the autism spectrum as a whole, their educational experiences, and whether they receive the help they may need to complete a degree or certificate or to advance to a 4-year college. Understanding who these youth are and maximizing their potential for college attendance is important for those who advise them.

Like all colleges in the US, 2-year colleges are required to offer reasonable accommodations to students with self-disclosed disabilities. Some interpret these accommodations to include help aimed at organizational and social skills needed for academic success [9, 10]. Two-year college support programs for students on the autism spectrum vary widely: from noncredit college experience programs with a residential component and classroom-based supports, to center-based disability support services delivered to degree-seeking students, to off-campus supports provided by outside agencies or family members [9]. Dual-enrollment transition programs allow some high school students to take classes at the community college while continuing to receive academic and social skills supports through an Individualized Education Program until the age of 21. The prevalence and need for various types of support are currently unknown.

College students on the autism spectrum may require services and accommodations due to impairments in executive functioning (such as inattentiveness and poor organizational skills), communication (such as maintaining the topic of conversation and comprehending abstract language), social interactions, and social relationships [11–13]. Additionally, psychiatric comorbidities are known to affect about 70% of children and adolescents on the autism spectrum [14, 15]. Challenges associated with psychiatric comorbidities can impact a student's ability to succeed in college and navigate the dynamics of the social environment [13, 16–18]. Still other students may have significant functional skills deficits that necessitate more intensive services to support the student on campus. Such students might attend non-degree granting programs that focus on vocational preparation and opportunities for social inclusion. Yet, to date, there is almost no research that elucidates the characteristics of which students on the autism spectrum typically attend different types of college settings and what supports they might require.

Known factors of postsecondary outcomes for young adults with autism include conversational abilities and functional impairments in addition to socioeconomic position [19, 20]. Parental expectation that the student will attend postsecondary education is an identified predictor of participation in postsecondary education for students with autism [21].

Experiences during high school may also be relevant factors in predicting whether students will go on for further education. For typically developing youth, a connection exists between participation in high school extracurricular activities and college-going [22]. However, little research has explored the effect of participation in extracurricular activities on outcomes for youth on the autism spectrum. Over 70% of high school youth on the autism spectrum engage in some type of extracurricular activity, whether volunteering, taking classes outside of school, church activities, or postschool activities, including youth with a wide range of conversational abilities [23]. Extracurricular activities may provide opportunities to build self-advocacy and self-determination skills, which are important for success within postsecondary education.

Participation in Individualized Educational Program (IEP) meetings within school may be another example of self-advocacy. While 85% of students with ASD have a transition plan [24], students with autism are half as likely as their peers with learning disabilities to actively participate in their own transition planning (IEP) meetings [25], particularly for those with greater cognitive and social skill deficits. In general, however, we understand little regarding factors that predict participation in postsecondary education or at 2-year colleges specifically.

In this exploratory study we use nationally representative data to conduct one of the first examinations of 2-year college experiences in young adults on the autism spectrum in the US. We examine the following questions. First, of young adults with autism who attend postsecondary education, what are the characteristics and educational experiences of those who solely attend 2-year college? Second, what are the differences in characteristics and educational experiences between those who attend 2-year college and those who attend 4-year college, who attend vocational-technical education, or who never attended postsecondary education of any type? Last, what types of services and/or accommodations do students with autism receive at 2-year colleges compared to those who attend 4-year college or vocational-technical education? Findings will help colleges plan for the growing number of students on the autism spectrum and will provide useful anticipatory guidance for youth, their families, high school counselors, and special education staff who facilitate transition planning.

## 2. Methods

### 2.1. Participants

We analyzed data from Wave 5 of the National Longitudinal Transition Study-2 (NLTS2), collected in 2009 when participants were aged 21–25 years. The NLTS2 provides one of the largest sources of prospective, population-based data on the postsecondary experiences of US students who received special education services during high school. Use of this data was approved by the US Department of Education (USDE) and deemed exempt by the Drexel University Institutional Review Board. Sample size reporting is rounded to the nearest 10 per agreement with the USDE.

The NLTS2 used a two-stage sampling plan. First, 500 local education agencies were randomly selected from across the US. Second, students who received special education services were sampled. This yielded 1,012 students who received special education services under the federal eligibility category of autism who were targeted for recruitment [26]. To ensure that results represented the target population, survey weighting was specifically calculated for each wave of data, and bias analysis was conducted at each wave as well [27, 28]. Results can be generalized to youth who were aged 13–17 and receiving special education services for autism at the start of the study in December 2000. More extensive discussion of NLTS2 methodology and questionnaire design is available [29].

Data analysis included students who received special education services under the special education categorical qualification of autism. At Wave 5, responses from 620 young adults who received special education services for autism during high school were available for analysis, yielding a retention rate of 67% at Wave 5 based on number of participants at Wave 1. For data presented in Table 1, rates of missing per covariate ranged from 0% to 17%. The highest rates of missing data were these variables: how well youth gets to places outside the home (17.4%), role of youth in the IEP setting (16.3%), household income (6%), and how well youth converses (6%). Given the branching logic of questioning in the survey, the number of possible participants who were asked to respond to questions regarding services and accommodations was relatively small. Therefore, results for Table 2 should be treated as exploratory findings. Missing data were not imputed for this descriptive study.

Individual school districts determined whether students qualified for special education under the category of autism in accordance with federal law [30]. We could not clinically verify autism diagnosis because these are secondary data. However, virtually all students who meet eligibility criteria for special education under the category of autism also meet DSM-IV-TR criteria for autism, using the diagnostic guidelines in use during the period of NLTS2 administration [31, 32].

### 2.2. Procedures

Data for this study came from NLTS2 interviews primarily conducted by telephone. Responses were obtained directly from the young adult to the extent possible, if parents reported that the young adult was capable of answering survey items either through telephone interview or a mailed survey. A parent or guardian responded to the survey questions in cases where the young adult was not able to complete an interview. The role of the investigators was limited to analysis of previously collected NLTS2 survey data.

### 2.3. Materials

Variables that represent student and family demographics, impairment characteristics, high school experiences, and parent expectation of postsecondary school attendance are presented in Table 1. Variables representing characteristics of students during high school included how well youth conversed, ability to get to places outside the home, whether youth participated in any extracurricular activities, and participation in the IEP process.

We measured frequency of participation in postsecondary education experiences with the survey items, “Since leaving high school, have you ever taken any classes from a 2-year, junior, or community college?”, from a “postsecondary vocational, business, or technical school,” or from a “4-year college or university.” Students were counted within only one postsecondary outcome: 2-year college only, 4-year college only, vocational/technical education but no college, or no postsecondary education. If the respondent answered yes to both 2-year college and vocational/technical school, follow-up questions differentiated the most appropriate designation. Young adults were also asked whether they felt that they had a disability, a precursor to receiving disability-related accommodations in the postsecondary education setting.

Variables related to help and services experiences are presented in Table 2. A sequence of questions assessed student service and support experiences during their postsecondary education. The first question, posed to all young adults, concerned use of help that was available to all students, “Did you ever get help with school work from this school like going to a tutor, or a study center, or a writing center?” If young adults responded affirmatively to having a disability, a follow-up question asked whether the school was aware of the disability or special need either before or after enrollment. Students who disclosed the disability to the school, and were therefore potentially eligible to receive accommodations, were then asked whether they received “any services, accommodations, or other help from the school to help you do your best there, like a note taker or more time to take tests because of a learning problem, disability, or other special need.” Those who responded affirmatively were asked, “What services, accommodations, or other help have you received there?” The NLTS2 interviewer coded the services, accommodations, and help that the student received.

Examples of categories and subcategories of services, accommodations, and help relevant to the needs of students on the autism spectrum are listed in Table 3. If the participant did not list any services, accommodations, or supports within the categories of human aides or out-of-classroom learning supports, follow-up prompts were asked: “Has there been any person assigned to help you, like a tutor, an interpreter, or someone who takes notes for you in class?” and/or “Have there been any supports for you outside of class, like a support group for students with disabilities or early registration?”

Students who received services, accommodations, or supports were next asked, “How useful have all the services, accommodations, and help with school work been in helping you stay in school and do your best there?” They were also asked whether they received enough services, accommodations, and help with schoolwork to do their best in school, as well as whether they had gotten any services or help on their own outside of the school.

### 2.4. Data Analysis

To understand the prevalence of postsecondary education experiences of youth with autism following high school, we estimated descriptive statistics for three groups of students to determine how many attended 2-year college only, 2-year and 4-year college, 4-year college only, vocational-technical education, or no postsecondary education. We examined postsecondary education attendance for these groups: (1) all youth with an autism spectrum disorder, (2) youth on the autism spectrum who ever attended postsecondary education of any type, and (3) youth on the autism spectrum who ever attended college. Those who attended both 2-year and 4-year colleges were excluded from further analysis due to inability to separate their experiences at each institution.

Our primary focus group for analysis was young adults on the autism spectrum who reported that they attended a 2-year college at some point since leaving high school but no other types of postsecondary schools. For purposes of contextualization and comparison, we also analyzed characteristics of young adults on the autism spectrum who experienced other postsecondary education outcomes: 4-year college, vocational/technical postsecondary education, and those who had never attended postsecondary education. We then compared postsecondary education and services experiences for those who attended a 2-year college to those who attended 4-year college, or vocational/technical education. Variables from Wave 1 were used to characterize students during high school (Table 1). Postsecondary outcomes and service experiences (Table 2) were measured using Wave 5 data. We used bivariate analyses and logistic regression to describe and test for group differences in characteristics of students during high school and in postsecondary education experiences, using young adults who attended 2-year college as the referent group for regression.

## 3. Results

Of all young adults on the autism spectrum represented in the NLTS2 at Wave 5, nearly one in five (17.7%) attended a 2-year college only, and an additional 9.2% attended both a 2-year and a 4-year college. Fewer students attended only a 4-year college (4.6%) or postsecondary vocational/technical education (7.4%). Most (61.1%) never attended any type of postsecondary education.

Of the 40% of young adults on the autism spectrum who attended postsecondary education of some type within the first eight years after high school, nearly 46% attended a 2-year college only; 11.7% attended a 4-year college only; 23.8% attended both 2- and 4-year colleges, and 19.1% attended a vocational/technical school. Of young adults on the autism spectrum who attended college, 85.6% ever attended a 2-year college, and 56.2% solely attended a 2-year college.

Students who reported that they attended a 2-year college only were primarily male (84.5%), White (85.3%), and non-Hispanic (97.6%) with a mean age of 23.2 years (range 21–25 years). Nearly 93% had at least one parent who attended any postsecondary education. Approximately half (47.1%) were from households with incomes in the $50,001–75,000 range. Significantly fewer 2-year college attendees were from households with incomes above $75,000 (21.4%) compared to those who attended 4-year colleges (47%, *p* < .05). Likewise, significantly fewer (12.1%) were from homes with incomes less than $25,000, compared to those who never attended postsecondary education (24.8%, *p* < .05).

Nearly 62% of students who attended 2-year college had little to no trouble conversing. Significantly more (63.5%, *p* < .01) of those who had no postsecondary education had a lot of difficulty or inability to hold a conversation. Most (64.6%) young adults who attended 2-year college reported doing “pretty well” to “very well” with functional skills like counting change, significantly more than students who never attended postsecondary education (36.8%, *p* < .05) but significantly fewer than those who attended 4-year college (97.1%, *p* < .01). Significantly more 2-year college students (85.7%) were able to navigate to places outside the home on their own than those who never attended postsecondary education (43.9%, *p* < .001) and those who attended vocational/technical school (61.5%, *p* < .01).

When asked at Wave 1, only 60.9% of parents of 2-year college students predicted that their youth would pursue postsecondary education, significantly higher than parents of those who never attended postsecondary education (16.5%, *p* < .001). Nearly all (93.8%) young adults who attended 2-year college participated in extracurricular activities during high school, significantly more than those who attended vocational education (74.4%, *p* < .001) or no postsecondary education (58.5%, *p* < .001). Nearly 66% provided some input in the IEP process or took leadership, significantly more than those who never attended postsecondary education (33.4%, *p* < .001).

Over half (56.1%) of students who attended only 2-year college took mostly vocational courses to train for a job, such as computer or business classes, as opposed to those who took mostly academic courses like English or science (26.1%), both vocational and academic (14.0%), or personal interest classes (3.8%). About 43% of 2-year college students utilized help with school work that was universally available to students, such as tutoring or writing lab. Other students used supports that were contingent upon disclosure of a disability.

Nearly half (48.6%) of those who attended 2-year college, and who disclosed their disability to the school, reported that they had received services, accommodations, or other help for their disability from the 2-year college. Further, 17.6% reported receiving services or help on their own outside of school. The most common accommodations for 2-year college students on the autism spectrum were human aides (62.5%), testing accommodations (56.4%), and physical adaptations in the classroom (34.3%) (Tables 2 and 3). Most students who attended 2-year college (87.3%) reported feeling that they had received enough services, accommodations, or help although only 68.0% reported that these services were somewhat or very useful.

## 4. Discussion

Continued education is an expectation for emerging adults in industrialized nations as a step along the pathway to a career [33]. In the US higher education is associated with higher incomes, better health, and improved quality of life [34]. Those with college degrees can expect to earn more and live longer lives [35, 36]. However, disparities in who attends college have persisted in the general population across the last half century, with nonattenders historically referred to as the “forgotten half” [37]. Public policy initiatives continue to attempt to close the gaps in college attendance and encourage college opportunities that include 2-year and community college programs.

The increase in youth with developmental disabilities, including autism spectrum disorder, who attend college is a relatively recent phenomenon, and there are few tracking mechanisms in place to tally the number of students who are accessing different postsecondary education options. This study found that over 85% of students on the autism spectrum who attend college in the US will enroll in a 2-year college at some point during the early years after high school. Well over half of these students solely attend a 2-year college.

It is critical for guidance counselors and transition planners to have useful predictors of which students might be candidates for 2-year college attendance. Overall, high school students who later attended a 2-year college could be characterized as having mid-range functional and communication abilities during high school relative to their peers on the autism spectrum with other types of postsecondary education outcomes. The conversational skills of young adults who attended 2-year college were generally of higher level than students who had no postsecondary education although students who attended 2-year college generally had greater levels of conversational impairment than their peers who had experiences at 4-year college. Similarly, they were able to navigate their community on their own and perform daily living skills more frequently than their peers in vocational/technical settings or those who had no postsecondary education but were not as adept as those who were able to attend 4-year college settings. This study also established that even students within the lowest conversational ability levels are accessing 2-year colleges.

Frequency of extracurricular participation and active participation in IEP transition planning activities was significantly higher in young adults who attended 2-year college compared to their peers in vocational education and those who did not attend postsecondary education. These indicators may be predictors of students who are candidates for 2-year college participation or may be viewed as transition experiences that could prepare a student for higher education settings.

Despite the finding that students with a wide range of abilities attended 2-year colleges, and the fact that all of these young adults required special education supports during high school, less than half of those who disclosed their disability to the 2-year college reported receiving any services, accommodations, or help. While the doors to 2-year colleges are open to students on the autism spectrum, gaps in supports may hinder their opportunities to succeed in this setting. Other studies have found lower rates of persistence and completion for students on the autism spectrum in 2-year settings [7], similar to students with other types of disabilities [38]. These issues must be addressed as more programs emerge that are designed to support these students in 2-year college programs.

While 2-year colleges are often touted as an avenue to success for students from disadvantaged backgrounds, students on the autism spectrum did not fit this characteristic. We found the majority of students on the autism spectrum who attended a 2-year college were from middle-to-upper income households with at least one parent who had some postsecondary education. The disadvantage these students experience appears to be primarily due to their disability, not their socioeconomic position.

Two-year college students on the autism spectrum in the US face significant gaps in access to supports for postsecondary education. Specialized college programs and supports offered for students with disabilities have increased in recent years due to federal support for comprehensive Transition and Postsecondary Programs for Students with Intellectual Disabilities (TPSIDs) authorized under the Higher Education Opportunity Act (HEOA) of 2008. However, these programs primarily serve students on the autism spectrum who have cooccurring intellectual disability, leaving students who are capable of accessing degree-seeking programs to pursue campus-based disability support services which are typically not tailored to the unique needs of students on the autism spectrum. Further, many TPSID programs are located on 4-year college campuses, not the 2-year/community college campuses which students on the autism spectrum frequently attend.

Issues of disability identity present another unique challenge in this population. College students must acknowledge and disclose a disability as a first step toward receiving disability support services in a college setting. Yet, one-third of college students on the autism spectrum who received special education during high school do not view themselves as having a disability or special need during young adulthood [39]. In cases where students are in need of help but are not receiving it, lack of identification of oneself as a student with a disability may signal an important barrier to receipt of disability support services. There is little research to explain lack of disability reporting among students on the autism spectrum. However, college students with other mental health disorders report feeling that they do not need services, lack time for services, or prefer to deal with issues on their own [40]. Nondisclosure may be related to stigma, limited student understanding of one's disability, or lack of resources [41]. Likewise, lack of service utilization by students with autism may be related to limited self-advocacy or self-determination skills, or not self-identifying a need for help [12, 42].

In summary, 2-year colleges are a frequent pathway in the transition to adulthood for many students in the U.S. and may offer a unique benefit for students on the autism spectrum. These settings serve as grounds for teaching and generalizing essential life skills including transportation navigation, employment, relationship-building, self-care, financial management, and sometimes residential living. Two-year colleges may function as a scaffold step between high school and independent living, offering a defined campus environment in which to develop life skills with potential support from fellow students who may be training to work with special needs populations. Yet, without adequate identification of students who may be candidates for 2-year programs, and corresponding improvements in access to the services they likely need for success in these institutions, this valuable option will be less than effective for helping young adults with autism to ultimately achieve higher quality lives.

## 5. Conclusions

Multiple opportunities exist for improving the success of students on 2-year college pathways beginning with enhancing planning for the transition to postsecondary education. Researchers note overall inadequate practices in transition planning [42], need for further research that explicitly connects transition planning interventions to intended outcomes [43], and a paucity of students with stated goals for transitioning into 2- or 4-year colleges [44]. Our findings of high rates of extracurricular activity participation during high school and active student participation in transition planning meetings indicate that many students who later go on to attend 2-year colleges do exhibit the seeds of self-determination skills that recent work has found to be related to positive postsecondary outcomes [45]. Further identification of which high school experiences and skills have the strongest relationship with positive postschool outcomes should be a primary research focus in the years ahead for informing transition practices.

Additional supports for parents of transition-age students may also be beneficial, as over one-fourth of families whose students attended 2-year colleges did not expect their child to attain this outcome. Parental expectations are contingent upon family awareness of available college options and support resources. A recent review of unmet needs of college students on the autism spectrum recommended further development of a student-in-training/parent-in-waiting model via dual-enrollment programs [46]. These programs would offer more explicit utilization of the student's IEP to develop supports in the college setting, gradual increase of ownership of academic and self-advocacy responsibilities to the student, and recognition that parents continue to play an important role in student support into the college years. Students and their families might also benefit from a focus on summer transition experiences between high school and 2-year college.

At the level of the postsecondary institution, few support programs are currently designed to address autism-specific needs of students. These needs may include highly specific guidance on understanding one's disability and related needs; navigating social situations and expectations; direct instruction in organizational systems; environmental accommodations for sensory integration dysfunction; and assistance for managing typical campus living skills including laundry, financial management, and sexual relationships [9].

This paper focused primarily on those who attended 2-year college to allow us to better examine student characteristics and support experiences in this setting alone. Findings from across our studies indicate differences in students who* only* attend a 2-year college versus those who* ever* attend a 2-year college. Although 9% of young adults with autism attended both 2-year and 4-year colleges, it was also not possible to accurately analyze their services experiences as these students may have had differing services, accommodations, or help across their 2-year and 4-year college settings. Wehman et al. (2014) [42] provide an extensive overview of the differences between 2- and 4-year settings and the needs of students who transfer from 2-year to 4-year programs.

### 5.1. Study Limitations

Study limitations were primarily related to the nature of the survey data. First, it was impossible to know whether students were enrolled in general or specialized postsecondary education programs such as TPSID-funded programs available on some college campuses. We were also unable to characterize important elements of educational quality identified by McEathron et al. (2013) including individualization of coursework, options for earning college credit, and degree of integration [47]. Third, while this study examined outcomes of those who were out of high school, we had no way to differentiate students who were able to access dual enrollment (in both high school special education and postsecondary education) during their transition-age years which may have provided some benefit to their postsecondary education outcomes. Finally, we were unable to accurately account for the potential effect of comorbid psychiatric diagnoses on postsecondary outcomes, as measures of medical diagnoses are limited in the NLTS2.

Despite these limitations, NLTS2 data offer an unprecedented glimpse into the postsecondary education outcomes and experiences of a large number of individuals on the autism spectrum in the US. Our findings indicate that students are accessing a variety of postsecondary education settings with a high prevalence of 2-year college experiences. Future research should delineate more specific needs of students in this setting and identify what supports are needed to improve persistence and completion rates as well as increase rates of transition into 4-year college programs.

## Figures and Tables

**Table 1:** Demographic and disability characteristics of students on the autism spectrum across postsecondary education outcomes. Percentage (95% confidence interval), test for difference versus the 2-year college column.

	2-year college	4-year college	Vocational/technical school	No postsecondary education
Male	84.5 (76.7, 92.4)	88.1 (75.8, 100)	84.2 (68.5, 99.9)	85.8 (81.8, 89.7)
Hispanic ethnicity	2.4 (0, 5.2)	4.2 (3.4, 5.0)	5.2 (0, 11.1)	12.1^*∗∗*^ (4.3, 19.9)
Race				
White	85.3 (79.6, 90.9)	73.9 (63.0, 84.8)	69.3^*∗*^ (57.0, 81.5)	54.8^*∗∗∗*^ (45.9, 63.7)
Black	8.3 (4.3, 12.2)	21.9^*∗∗*^ (11.4, 32.4)	15.8 (4.8, 26.8)	25.9^*∗∗∗*^ (22.7, 29.2)
Other race	6.4 (2.0, 10.9)	4.2 (3.0, 4.5)	14.9 (4.9, 24.9)	19.3^*∗∗*^ (10.3, 28.2)
Either parent attended any postsecondary education	92.7 (87.2, 98.3)	100^*∗∗∗*^ (100, 100)	78.5 (58.6, 98.3)	66.5^*∗∗∗*^ (60.0, 73.0)
Parent or guardian household income				
Up to $25,000	12.1 (4.4, 19.7)	8.8 (2.3, 15.2)	11.0 (5.2, 16.8)	24.8^*∗*^ (19.1, 30.5)
$25,001–$50,000	19.4 (10.1, 28.7)	15.3 (2.6, 27.9)	32.1 (14.1, 49.7)	27.9 (22.3, 33.5)
$50,001–$75,000	47.1 (37.5, 56.8)	28.9 (15.2, 42.7)	28.5 (7.6, 49.3)	35.1 (28.9, 41.4)
More than $75,000	21.4 (14.8, 28.1)	47.0^*∗*^ (28.4, 65.6)	28.5 (11.2, 45.8)	12.1^*∗*^ (7.9, 16.3)
Characteristics of youth during high school				
How well youth converses				
Has a lot of trouble or not able to converse at all	38.5 (25.7, 51.4)	10.9 (7.3, 14.4)	39.3 (18.1, 60.5)	63.5^*∗∗*^ (54.7, 72.2)
Little trouble	52.1 (39.5, 64.7)	61.5 (48.7, 74.3)	35.4 (17.6, 53.1)	30.4^*∗∗*^ (22.4, 38.3)
No trouble	9.4 (4.6, 14.1)	27.7^*∗∗*^ (13.9, 41.4)	25.4^*∗*^ (4.8, 45.9)	6.2 (2.8, 9.5)
Counts change pretty or very well	64.6 (43.4, 85.7)	97.1^*∗∗*^ (90.4, 100)	64.2 (49.0, 79.4)	36.8^*∗*^ (30.0, 43.7)
Gets to places outside the home pretty or very well	85.7 (78.6, 92.8)	82.8 (74.2, 91.5)	61.5^*∗∗*^ (47.3, 75.8)	43.9^*∗∗∗*^ (35.0, 52.9)
Parent expected youth to probably or definitely attend postsecondary school	60.9 (48.0, 73.7)	84.8 (80.6, 89.1)	53.5 (30.7, 76.2)	16.5^*∗∗∗*^ (10.6, 22.4)
Any extracurricular activities	93.8 (89.8, 97.7)	90.3 (85.3, 95.3)	74.4^*∗∗∗*^ (58.6, 90.1)	58.5^*∗∗∗*^ (52.0, 65.0)
Participated in IEP or transition planning during high school (student provided some input or took leadership)	65.5 (55.6, 75.4)	82.7 (74.5, 90.9)	50.5 (31.0, 70.1)	33.4^*∗∗∗*^ (27.6, 39.2)

Note: source: National Longitudinal Transition Study-2, Waves 1 and 5. Weighted to population levels. Variances adjusted for sampling method. Logistic regression analysis used 2-year college as the referent group; ^*∗*^ *p* < .05, ^*∗∗*^ *p* < .01, and ^*∗∗∗*^ *p* < .001.

**Table 2:** Postsecondary education supports and services experiences of youth on the autism spectrum. Percentage (95% confidence interval), test for difference versus the 2-year college column.

	2-year college	4-year college	Vocational/technical school
Attendance and coursework at postsecondary institution			
Continuity of attendance			
Enrolled continuously	80.4 (67.8, 93.0)	93.6 (83.2, 100)	78.1 (66.8, 89.5)
Full- or part-time student as of most recent term			
Full time (≥12 hours' credit)	50.4 (36.5, 64.3)	86.2^*∗∗∗*^ (77.7, 94.7)	78.4^*∗*^ (60.4, 96.3)
Universally available student supports			
Ever accessed help with school work available to all students at the postsecondary institution	43.2 (30.2, 56.2)	41.8 (35.0, 48.5)	20.3^*∗*^ (15.4, 25.3)
Received services or help on his/her own outside of school	17.6 (8.3, 26.9)	22.9 (7.5, 38.3)	17.5 (8.3, 26.6)
Disability-related services			
Young adult considers self to have a disability	70.2 (58.2, 82.1)	63.6 (39.9, 87.4)	80.6 (70.4, 90.0)
School was aware of disability either before or after enrollment	69.1 (57.9, 80.2)	62.7 (55.0, 70.4)	99.0^*∗∗*^ (96.6, 100)
Received any services, accommodations, or other help	48.6 (34.1, 63.1)	30.0 (18.5, 41.5)	65.5 (48.8, 82.3)
Type of accommodations or services received			
Human aides	62.5 (51.4, 73.7)	70.4 (39.3, 100)	44.9 (19.0, 70.9)
Testing accommodations	56.4 (40.9, 71.8)	84.2^*∗*^ (75.4, 92.9)	44.6 (16.2, 73.0)
Physical adaptation	34.3 (14.1, 54.4)	49.9 (25.5, 74.3)	13.1^*∗*^ (10.0, 16.1)
Out-of-classroom learning supports	19.8 (0, 41.3)	25.0 (19.6, 30.3)	19.5 (14.9, 24.1)
Materials/technology adaptations	16.0 (6.6, 25.4)	58.7^*∗∗*^ (24.3, 93.1)	—
Accommodations in assignments	14.4 (6.1, 22.6)	14.7 (2.1, 27.4)	27.2 (20.7, 33.6)
All supports and services			
Received enough help, services, or accommodations in and out of school	87.3 (78.5, 96.1)	91.2 (82.4, 100)	93.3 (—, —)
Help, services, and accommodations received in and out of school were somewhat or very useful	68.0 (52.8, 83.3)	55.5 (28.1, 82.8)	—

Note: source: National Longitudinal Transition Study-2, Wave 5. Weighted to population levels. Variances adjusted for sampling method. Cell sizes that are too small to report are denoted (—). Logistic regression analysis used 2-year college as the referent group; ^*∗*^ *p* < .05, ^*∗∗*^ *p* < .01, and ^*∗∗∗*^ *p* < .001.

**Table 3:** Examples of services, accommodations, or other help received during postsecondary education by primary categories and subcategories.

Testing accommodations	More time to take tests
	Having tests read aloud
	Scribe to record answers
	Different tests
	Different grading standards
	Different setting to take tests

Assignment accommodations	Additional time to finish assignments
	Shortened length of assignments

Materials technology adaptations	Use of computer or spell checker in class or to take tests
	Special use of calculator, listening/recording devices, tape recorder
	Provision of written materials (e.g., copies of lectures, outlines, course notes)
	Books on tape
	Computer software designed for students with disabilities

Human aides	A reader or interpreter
	Note taker in class
	Personal aide or instructional assistant to help you in class
	Tutor
	Support person to monitor academic progress or help with managing workload

Out-of-classroom learning supports	Behavior management program
	Help with learning strategies or study skills
	Support group for students with disabilities
	Early registration

Physical adaptations in classroom	Physical changes to the classroom, special desks
	Changes to equipment, like different lab equipment in a science class

Note: source: National Longitudinal Transition Study-2, Wave 5. Youth Interview.
